# Commentary on “Extensive study on the associations of 12 composite inflammatory indices with colorectal cancer risk and mortality: a cross-sectional analysis of NHANES 2001–2020”

**DOI:** 10.1097/JS9.0000000000004548

**Published:** 2025-12-11

**Authors:** Ximeng Ren, Yao Song, Yang Liu, Dapeng Ding

**Affiliations:** Department of Clinical Laboratory, The First Affiliated Hospital of Dalian Medical University, Dalian, China

*Dear Editor,*

We read with great interest the recent article by Zhou *et al*^[1]^, which systematically evaluated the associations between 12 inflammatory indices and colorectal cancer (CRC) risk and mortality using NHANES data. The study highlights the potential of easily accessible biomarkers such as neutrophil-to-lymphocyte ratio (NLR) and lymphocyte-to-monocyte ratio (LMR) in stratifying CRC risk. While this work provides valuable insights into the clinical application of inflammatory markers, its methodological approach prompts several considerations. This correspondence adheres to the TITAN guidelines^[2]^.

In addressing the issue of multiple comparisons, this study mitigates its impact through multi-model adjustments and cautious interpretation, yet it does not employ rigorous statistical correction methods (such as Bonferroni or false discovery rate), thereby retaining a risk of false positives. As an exploratory investigation, this approach helps avoid excessive correction that could lead to false negatives but, to some extent, compromises the robustness of the inferences. We suggest that subsequent confirmatory studies explicitly adopt multiple comparison corrections or, at minimum, address this aspect in the limitations section to enhance the reliability of the findings^[3]^.

Furthermore, the study defines the control group as all non-CRC participants, a population that includes individuals with other inflammatory diseases or cancers. This may introduce significant confounding and dilute the true association between inflammatory markers and CRC, potentially leading to an underestimation of effect sizes. To more accurately elucidate the specific relationship between inflammatory indicators and CRC, future analyses could consider establishing a more refined control group – for instance, by excluding participants with autoimmune disorders, other inflammatory conditions, or other cancers – or by applying propensity score matching to strengthen the conclusiveness of the results^[4]^.

Last, we wish to raise a point regarding the study design and interpretation. The cross-sectional design of this study measures prevalence ratios, which are interpreted as “risk” in the text – a conceptual nuance that may warrant further clarification. A critical, yet overlooked, limitation is that inflammatory markers were measured after cancer diagnosis, and their levels could be influenced by tumor stage, prior treatments (such as surgery or chemotherapy), and survival status. Consequently, it remains challenging to fully disentangle whether the observed alterations in inflammatory profiles represent a cause of cancer development or are instead a consequence of disease progression or treatment. This uncertainty may affect the robustness of directly inferring NLR, LMR, and other markers as early screening biomarkers, and we recommend that it be comprehensively considered in subsequent research and clinical interpretation.

In summary, we respectfully offer these methodological reflections to contribute nuanced perspectives to this field. While this study provides a systematic evaluation of inflammatory markers in CRC with considerable exploratory and clinical value, we encourage future work to build upon this foundation through prospective designs, refined control groups, and clinical utility assessments – ultimately advancing these biomarkers toward translational application and higher-level evidence for CRC management.

## Data Availability

Not applicable.
